# INTEREST GROUP SESSION—GRANDPARENTS AS CAREGIVERS: GERONTOLOGICAL INTERVENTION SCIENCE: FOUR DIFFERENT APPROACHES TO GRANDFAMILY RESEARCH

**DOI:** 10.1093/geroni/igz038.1291

**Published:** 2019-11-08

**Authors:** Christine Fruhauf, Loriena Yancura, Bert Hayslip

**Affiliations:** 1 Colorado State University, Fort Collins, Colorado, United States; 2 University of Hawaii at Manoa, Honolulu, Hawaii, United States; 3 University of North Texas, Denton, Texas, United States

## Abstract

For nearly three decades, the interest in custodial grandparents and their grandchildren has resulted in empirical studies from scholars addressing both the challenges and strengths often experienced by grandfamilies (i.e., grandparents and the grandchildren in their home). As the scholarship of intervention science increases in interest and as scholars continue to advance the field of knowledge with grandfamilies, it is important to share best-practices as we plan the future of gerontological intervention science. In this symposium, investigators with extensive experience researching custodial grandparents and in creating, testing, and refining interventions with grandfamilies will present theoretical, methodological, and practical lessons learned and empirical findings from their interventions. The first paper, by Musil and colleagues, will address a current online training program addressing resourcefulness with grandmothers. Second, Montoro-Rodriguez and Hayslip will discuss the use of the selective optimization with compensation model as applied to a goal-setting intervention with grandparents. The third paper by Fruhauf, Yancura and colleagues will address the progress and impact from the first two years of their intervention with grandparents, grandchildren, and service providers. The final paper, presented by Webster, Smith, and Infurna will focus on a dyadic intervention with grandparents and adolescent grandchildren as they seek to build social intelligence. Hayslip, the discussant, will integrate key points from these interventions while addressing considerations for future research as scholars embark on refining and testing their interventions related to grandparents raising grandchildren.

